# Optimization of Time-Course Experiments for Kinetic Model Discrimination

**DOI:** 10.1371/journal.pone.0032749

**Published:** 2012-03-05

**Authors:** Nuno F. Lages, Carlos Cordeiro, Marta Sousa Silva, Ana Ponces Freire, António E. N. Ferreira

**Affiliations:** 1 Centro de Química e Bioquímica, Departamento de Química e Bioquímica, Faculdade de Ciências da Universidade de Lisboa, Lisboa, Portugal; 2 Department of Pharmacology, Green Center for Systems Biology, University of Texas Southwestern Medical Center, Dallas, Texas, United States of America; Technical University of Madrid, Italy

## Abstract

Systems biology relies heavily on the construction of quantitative models of biochemical networks. These models must have predictive power to help unveiling the underlying molecular mechanisms of cellular physiology, but it is also paramount that they are consistent with the data resulting from key experiments. Often, it is possible to find several models that describe the data equally well, but provide significantly different quantitative predictions regarding particular variables of the network. In those cases, one is faced with a problem of model discrimination, the procedure of rejecting inappropriate models from a set of candidates in order to elect one as the best model to use for prediction.

In this work, a method is proposed to optimize the design of enzyme kinetic assays with the goal of selecting a model among a set of candidates. We focus on models with systems of ordinary differential equations as the underlying mathematical description. The method provides a design where an extension of the Kullback-Leibler distance, computed over the time courses predicted by the models, is maximized. Given the asymmetric nature this measure, a generalized differential evolution algorithm for multi-objective optimization problems was used.

The kinetics of yeast glyoxalase I (EC 4.4.1.5) was chosen as a difficult test case to evaluate the method. Although a single-substrate kinetic model is usually considered, a two-substrate mechanism has also been proposed for this enzyme. We designed an experiment capable of discriminating between the two models by optimizing the initial substrate concentrations of glyoxalase I, in the presence of the subsequent pathway enzyme, glyoxalase II (EC 3.1.2.6). This discriminatory experiment was conducted in the laboratory and the results indicate a two-substrate mechanism for the kinetics of yeast glyoxalase I.

## Introduction

Mathematical modeling is a key tool to investigate how the dynamics of biochemical systems emerges from the interactions of cellular molecular components [1]. The reliability of the predictions derived from a model based on ordinary differential equations (ODE) often depends on finding accurate parameter values and selecting the most appropriate network structure and rate equations. Parameter estimation and model discrimination are, therefore, two main concerns in Systems Biochemistry. To solve these problems, several conditions must be fulfilled. For instance, it is paramount that a minimal set of variables can be experimentally observed to ensure parameter identifiability, meaning that the parameters of the model can be uniquely estimated. Often, despite satisfactory parameter estimation, the selection of the best model from a set of candidate models is not clear from the experimental data available *a priori*. In such cases, one possible strategy is to design experiments specifically to discriminate which model better explains the observed behavior of the investigated biochemical system. This paper focuses on the implementation of this strategy, assuming that (i) the observable variables to be measured were already chosen (possibly due to experimental constraints concerning which biochemical variables can actually be measured) and (ii) estimates for the parameters of the candidate models were previously obtained. A procedure is presented to optimize time-course kinetic experiments so that the divergence between the time courses predicted by the models under consideration is maximized. In these conditions, the relative competence of the candidate models in describing new experimental data, according to appropriate statistical criteria, should be clear. This idea has been explored before for two candidate-model problems [2], [3], [4], [5]. These works share the common feature that a distance between quantitative predictions drawn from the models (often the weighted sum of the squared differences between outputs computed over the time courses predicted by each model) is maximized to find the optimal experimental conditions. They differ in the experimental parameters and manipulations considered in the design of the discriminatory experiments: the optimal spacing in the time between measurements [5], the perturbation applied to a running biochemical system and the optimal instant for such perturbation [3] or different combinations of constant or sinusoidal input variable values [2]. The measure used to choose the best model also differs among the different approaches and ranges, from simple *L*_2_ distances in the amplitudes [4] or phases [2] of the model outputs, to the fitting scores to new data generated at the discriminatory conditions [2]. The goal of finding experimental conditions such that the predictions of the models are sufficiently different to allow discrimination regardless of measurement noise was explicitly stated in one of these works [4].

In our study, we follow the general idea of finding constant inputs that maximize the difference between the predicted time courses of concentrations as model outputs. The model divergence metric used here is based on the Kullback-Leibler distance, a measure of the difference between two probability distribution functions [6], [7], as defined in equation 1.
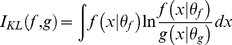
(1)In this equation, *f* and *g* are probability density functions, *x* is the vector of observable variables, *θ_f_* and *θ_g_* are the vectors of parameters of *f* and *g* respectively, and the integral is computed over the domain of the distributions. *I_KL_*(*f*, *g*) is a measure of how well distribution *g* approximates distribution *f*. *I_KL_*(*f*, *g*) is not a symmetrical distance, since distribution *g* may approximate distribution *f* better than distribution *f* approximates *g*.

An extension of the Kullback-Leibler distance to the space of positive functions was proposed [8], according to the equation 2.
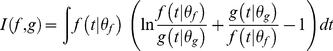
(2)This measure of divergence was used in the context of nonlinear regression for the estimation of pharmacokinetic parameters as an alternative to ordinary least squares and extended least squares [8]. Its form is derived from the application of a Minimum Relative Entropy Principle to nonlinear estimation problems. In estimation, *f* represents experimental data and *g* the values predicted by a model to be fit. Simulations for a combination of typical pharmacokinetic functions with different error models showed that minimization of this distance function to estimate parameters has a performance comparable to the extended least squares method and that it only performed poorly for constant error rate problems [8]. However, to apply this measure of divergence, a particular measurement error model does not need to be considered or postulated. This was concluded to be one of the main advantages of using function *I* (*f*, *g*) in estimation problems [8].

We suggest the use of the extended Kullback-Leibler distance, *I* (*f*, *g*), as a measure of divergence between biochemical kinetic ODE based models describing the time variation of the concentrations variables. In this context, *f* and *g* in equation 2 are the time courses predicted by kinetic models for the experimentally measurable variables, the observable model outputs. For model discrimination, the experimental conditions that maximize *I* (*f*, *g*) computed over the time courses predicted by every pair of candidate models *f* and *g*, in both directions, are considered to be optimal for an experiment aiming at the selection of one candidate model. By choosing this measure of divergence and given its statistical properties in estimation problems, we follow the idea of maximizing the difference between predictions of models to an extent such that discrimination can be achieved despite measurement errors [4].

As an illustration of the use of such measure, we designed an experiment to discriminate between two kinetic models proposed for the yeast glyoxalase system that differ in the kinetics of the first enzyme of the pathway, glyoxalase I, one being a single-substrate model and the other a two-substrate model. The glyoxalase pathway (comprising glyoxalase I and glyoxalase II) is responsible for the elimination of methylglyoxal, a toxic, mutagenic and highly reactive metabolite present in all living cells. Methylglyoxal is formed mainly as a non-enzymatic by-product of glycolysis [9]. This system is particularly important in diabetes and in neurodegenerative disorders (like familial amyloidotic polyneuropathy, Alzheimer's and Parkinson's diseases), since it prevents the formation of methylglyoxal-derived advanced glycation end-products involved in these diseases [10], [11], [12], [13]. Glycation changes protein structure with consequent loss of function, but notably in chaperones like α-crystallin [14] and fibrinogen [15] may also potentiate activity.

The kinetic mechanism of glyoxalase I has been a matter of debate for years as this enzyme acts upon a mixture of three substrates: methylglyoxal, glutathione and the hemithioacetal resulting from the non-enzymatic reaction of the first two.

In this work we address this question by implementing a discriminatory experiment leading to the comparison of the predicted time courses from each model with laboratory data that allowed the conclusive selection of the two-substrate model.

## Results and Discussion

### Multi-optimization framework

**Figure 1:**
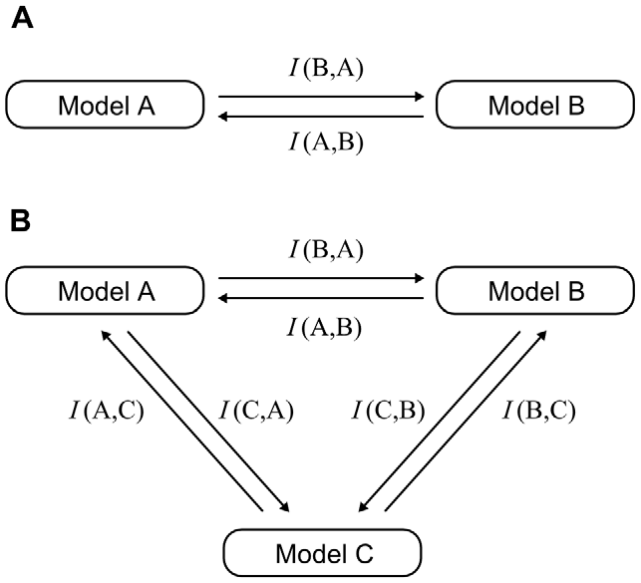
Discrimination of kinetic models by maximization of the extended Kullback-Leibler distance (*I *). Conditions are sought that maximize *I* in both directions between any two models. In a two candidate model scenario (A) two functions must be simultaneously optimized. In a three candidate model scenario (B) six functions must be simultaneously optimized. After optimization, the set of solutions approximate a Pareto front and represent a compromise between the various objectives in the sense that, for any solution, the value of any objective could only be increased if the value of another objective was simultaneously decreased.

The extended Kullback-Leibler distance *I* (*f*, *g*) is a directed measure and must be maximized in both directions even in the simplest two-model case, as summarized in figure 1A, requiring a multi-objective optimization approach. An alternative would be the optimization of the sum of *I* (*f*, *g*) and *I* (*g*, *f*). However, simultaneous maximization in both directions is preferable, as maximizing the sum may favor maximization in one direction at the expense of the other. Some multi-objective optimization problems may be solved by assigning different weighting factors to each objective according to their relative importance and using a single-objective optimization algorithm. In this case, however, objectives have equal importance – all candidate models should be tested in conditions which do not favor the selection of any model.

As opposed to single-objective optimizations, in multi-objective problems several solutions of equivalent quality can be found, each with different trade-offs regarding the scores for the different objectives. The set of the optimal solutions for a multi-objective optimization problem is the “Pareto front” [16], [17], [18]. The approximation of solutions to the Pareto front may be compared by the dominance criterion, [17], [19]: a solution *u* dominates a solution *v*, i.e. 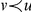 if and only if condition 3 is verified:
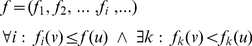
(3)


The optimal solutions are those for which the score of one objective cannot be improved without decreasing the score of other objectives. Therefore, optimal solutions are non-dominated. The user should choose among the final non-dominated solutions resulting from the algorithm those that provide a feasible design, taking experimental constraints into consideration.

### Application example

The concept of Pareto optimality and the extended Kullback-Leibler distance were combined to find an optimal experimental design for model discrimination. The procedure was applied to optimize an experiment to discriminate between two kinetic models proposed for the yeast glyoxalase system that differ in the kinetics of the first enzyme of the pathway (glyoxalase I).

**Figure 2:**
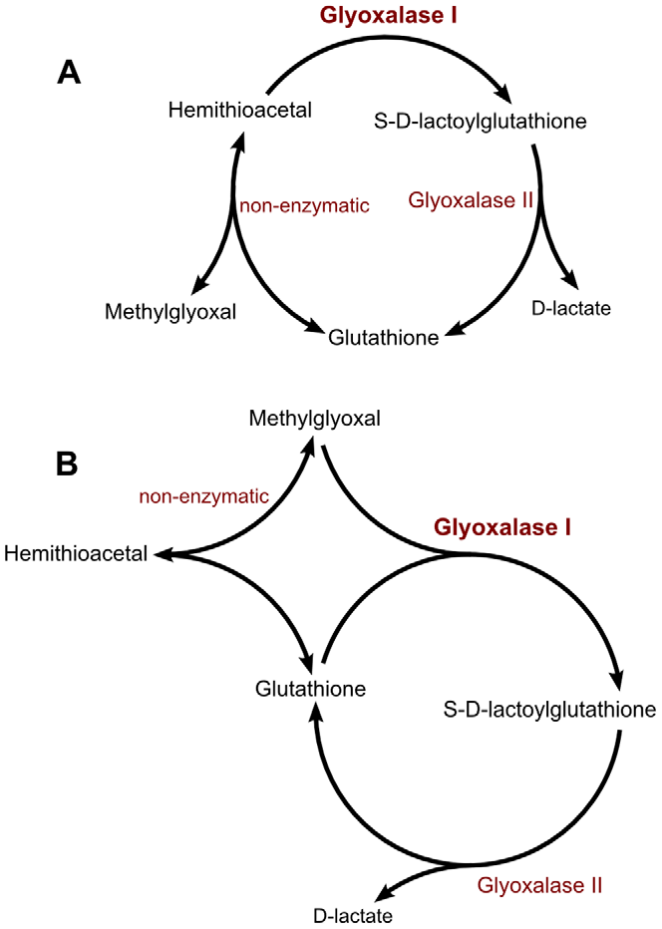
Kinetic models of the glyoxalase pathway. In model 1 (A), glutathione (GSH) and methylglyoxal (MGO) form a hemithioacetal (HTA) which is the substrate of glyoxalase I. In model 2 (B), glutathione and methylglyoxal are sequential substrates of glyoxalase I and the hemithioacetal is formed at the active centre of the enzyme. Glyoxalase II is a one-substrate-one-product irreversible Michaelis Menten enzyme, catalyzing the hydrolysis of *S*-D-lactoylglutatione (SDLGS) into D-lactate (D-Lac) and glutathione. The rate laws assumed in the models are expressed in equations 15 to 18.

In both models, shown in figure 2, the substrates of the pathway, glutathione (GSH) and methylglyoxal, undergo a non-enzymatic condensation that results in the formation of a hemithioacetal. This non-enzymatic step precedes the reactions catalyzed by the enzymes of the pathway, a feature that is quite uncommon in biochemical networks. In model 1, glyoxalase I is a one-substrate Michaelis-Menten irreversible reaction that catalyzes the isomerization of hemithioacetal [20]. In model 2, glyoxalase I binds methylglyoxal and glutathione directly and competes with the non-enzymatic step for these substrates [21]. The product of glyoxalase I is the adduct *S*-D-lactoylglutathione (SDLGS). Glyoxalase II acts downstream of glyoxalase I, converting SDLGS into GSH and D-lactate, and is widely accepted to follow irreversible one-substrate Michaelis-Menten kinetics [22], [23], [24], [25], [26], [27], [28], [29], [30].

The difficulty in selecting among the two models for the human glyoxalase I led to the proposal of a branched mechanism of which models 1 and 2 are particular cases [31], [32]. Nevertheless, this broader model, which was consistent with initial rate experiments, did not come into much use in subsequent works since authors generally choose the one-substrate model [27], [28], [33], [34], [35] over the two-substrate model [29].

In order to complement the theoretical experimental design procedure with an actual laboratory experiment, we restricted the design to the maximization of the difference of output variable SDLGS. In contrast to the other concentration variables, which can only be measured by analytical derivatization methods performed at discrete time points, the concentration of this variable can be easily determined by following its absorbance on a UV-visible spectrophotometer with high frequency sampling (above 1 Hz).

The concentrations at time zero are the most obvious and easiest experimental variables controllable by the user. We considered the initial substrate concentrations as variables to be optimized in model discrimination.

### Parameter estimation

Before a model discrimination experiment is designed, it is assumed that every candidate model is equally adequate to describe previous experimental observations and that their parameters have been estimated. Only after all models are fully characterized, can a strategy be sought to find an experimental setup for which the divergence between the predictions of any pair of candidate models is simultaneously maximized. A model is considered to be fully characterized when the network structure, the reaction rate laws and the kinetic parameters are all known. In turn, this means that the corresponding ODE equations have no unknown functions or constants in their mathematical expression apart from the dynamic variables.

The goal, in the application example, was the discrimination between two models for the yeast glyoxalase pathway that differ only in glyoxalase I kinetics. However, for the design of the discriminatory experiment we considered the presence of glyoxalase II. The kinetics of this second enzyme had also to be parameterized and, for this purpose, we used glyoxalase II from bovine liver, which is commercially available. This enzyme is described by the same rate equation in both models and fulfils the key role of regenerating glutathione as the pathway cofactor.

**Figure 3:**
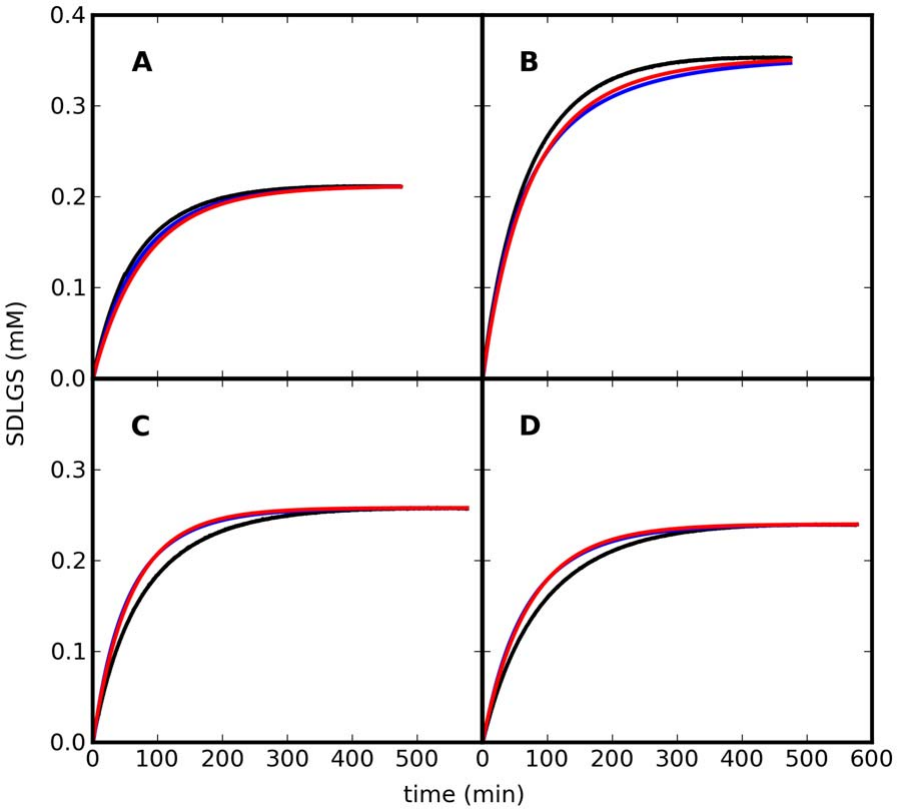
Time courses of SDLGS formation in the reaction of yeast glyoxalase I. Black: experimental data. Blue: time course predicted by model 1. Red: time course predicted by model 2. Experimental time courses and initial concentrations are included in dataset S1.

The kinetic parameters, determined from a collective fit to the time courses included in dataset S1, are shown in table 1. Figure 3 shows the data used for parameter estimation associated with glyoxalase I, along with the predictions by the two candidate models for the time course of the product SDLGS in the absence of glyoxalase II. Some of the parameters are associated with rather large standard errors (table 1). This is commonly observed in time-course collective fits or when the number of measured variables is too few, a phenomenon previously described as *sloppiness* [36]. However, it has been found that even in models exhibiting sloppiness, as indicated by the large standard errors, the predictability of the models remains acceptable as similar time-course responses are predicted over a wide parameter variation [36]. This was actually observed in our results, as shown in figure 3: the experimental time courses of SDLGS are very close to the time courses predicted by each of the two candidate models. As a consequence, the discrimination between the two proposed models of the glyoxalase I from data generated using glyoxalase I alone is a difficult problem. In these simple assays, the non-enzymatic formation of hemithioacetal and the reaction of glyoxalase I are the only reactions occurring, without regeneration of the cofactor glutathione. It should be noted that the initial substrate and enzyme concentrations used in this parameterization are rather representative since they are used in standard protocols to assay glyoxalase I activity [29]. In spite of their large standard errors, the estimates for the parameters were used subsequently as nominal values for the model discrimination procedure.

**Table 1:** Kinetic parameters for *S. cerevisiae* glyoxalase I and bovine liver glyoxalase II.

Enzyme	Parameter	Value ± Standard error
**Glyoxalase I, model 1**	*k*_cat GLOI,1_	(8±3.0)×10^4^ min^−1^
	*K*_m,HTA_	(0.2±0.14) mM
**Glyoxalase I, model 2**	*k*_cat GLOI,2_	(1.7±0.38)×10^5^ min^−1^
	*K*_m,GSH_	(0.87±0.11) mM
	*K*_m,MGO_	(1.2±0.19) mM
**Glyoxalase II**	*k*_cat GLOII_	(3±2.2)×10^2^ min^−1^
	*K*_m SDLGS_	(3±2.1) mM
**Non-enzymatic reaction**	*k_f_*	0.34 mM^−1^ min^−1^
	*k_r_*	1.01 min^−1^

Parameters were estimated by collective fit to time-course data, as detailed in Methods, except for the non-enzymatic reaction rate constants, for which previously reported values were used [29]. Dataset S1 includes four time courses used in the estimation of glyoxalase I parameters and four time courses used in the estimation of glyoxalase II parameters.

### Model discrimination

The global optimal solutions of the optimization problem might not be usable due to specific experimental limitations: for instance, optimal substrate concentrations may lead to intermediate concentrations below the limit of detection or above the measurable range; the necessary amount of reagents may be so high that the experiment would be extremely expensive or not feasible due to solubility issues. Therefore, appropriate allowable ranges were assigned to the variables to be optimized.

In the glyoxalase system, the activities of the two enzymes are commonly assayed by following the intermediate SDLGS at its maximum absorption wavelength (240 nm) with an absorption coefficient of 2.86 mM^−1^ cm^−1^ [37]. For the initial concentrations of glyoxalase I and II, 2.0×10^−3^ mM and 4.0×10^−4^ mM were set as upper bounds for the optimization, respectively. These limits were chosen so that several replicate experiments could be performed from single commercial enzyme batches. For the substrates glutathione and methylglyoxal, boundaries for the concentrations were set to 1 mM, to ensure that the changes of SDLGS signal were within the spectrophotometer range. These boundary values are summarized in table S1.

The performance of the extended Kullback-Leibler distance was compared with two other measures of model divergence used in previous works:

1- Simple *L*_2_-norm (non-weighted)
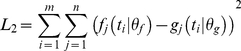
(4)
2- The *L*_2_-norm weighted by the square of the mean values of model variables [2]

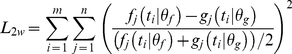
(5)

The expression for the Kullback-Leibler distance extended to the space of positive functions (equation 2) [8], using a discrete version for equidistant time points, is:
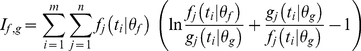
(6)In equations 4, 5 and 6, *f_j_* (*t_i_*|*θ_f_*) and *g_j_* (*t_i_*|*θ_f_*) are the values of variable *j* at time point *i* predicted by models *f* and *g*, respectively. The system has *n* observable variables and the time course has *m* time points.

**Figure 4:**
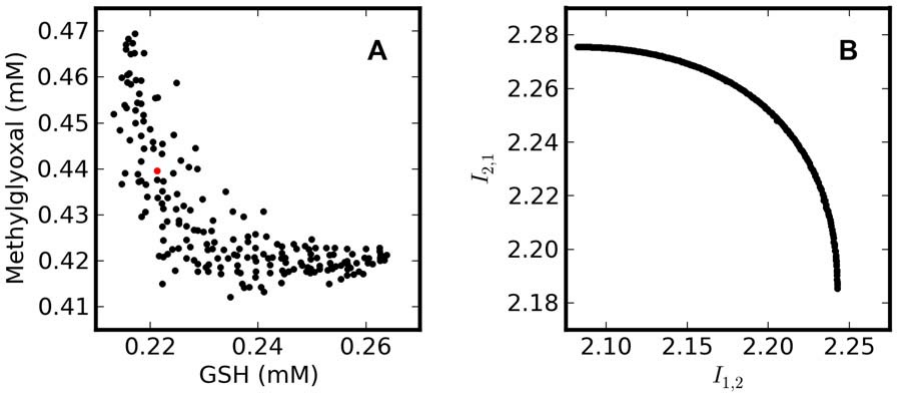
Optimization of experimental design for model discrimination. A - Optimal initial concentrations of methylglyoxal and glutathione (solutions approximating the Pareto front) for the discrimination of the two models presented in figure 2. B – Corresponding values of the extended Kullback-Leibler distances (optimization objectives); Concentration of glyoxalase I is 2.0×10^−3^ mM and concentration of glyoxalase II is 4.0×10^−4^ mM. The red dot indicates the initial concentrations used in the discriminatory experiment.

Both *L*_2_ norms were ineffective for this problem since the optimization converged to the bounds of the allowed ranges for the concentrations, both for the initial substrates and the enzymes. The use of the extended Kullback-Leibler distance (equation 6) required the implementation of a multi-objective optimization strategy (figure 1A). With this metric, convergence to optimal substrate concentrations was achieved, although enzyme concentrations converged to the upper-bound limits. This means that using this divergence measure, it was possible to optimize the substrate concentrations for a discrimination experiment if the enzyme concentrations were set to constant values. Running the optimization while removing enzyme concentrations as parameters to be optimized resulted in a set of solutions approximating a Pareto front for the initial values of glutathione and methylglyoxal. The solutions which approximate the Pareto front are shown in figures 4A (in the space of the solutions) and 4B (in the space of the objective functions) and were obtained after termination of the optimization by the maximal generation number criterion.

The optimal solutions have a little spread over the solution space (within 10% of average value for methylglyoxal and 6% for GSH) and, as a consequence, the time courses predicted by each model are very similar.

**Figure 5:**
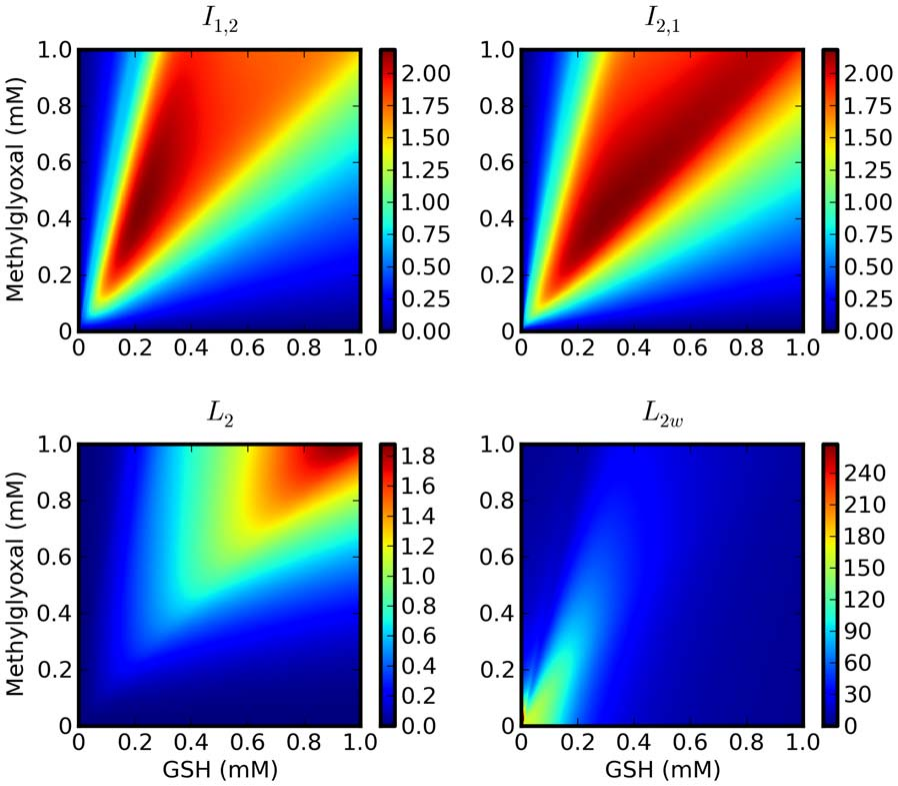
Landscapes of different measures of model divergences in the allowed optimization range of concentrations of pathway substrates. Measures of model distances are *I*_1,2_ : extended Kullback-Leibler distance of model 2 from model 1 (equation 6). *I*_2,1_ : extended Kullback-Leibler distance of model 1 from model 2 (equation 6). *L*_2_ : simple *L*_2_ norm (equation 4). *L*_2*w*_ : weighted *L*_2_ norm (equation 5).

The landscape of optimization objectives is shown in figure 5. The two directed extended Kullback-Leibler distances between the two models both have a clear region containing a maximum (or maxima) and the multi-objective optimization gave solutions that took into account both objectives. In the case of the *L*_2_ norms, the landscape explains why the single objective maximization of these functions failed to provide solutions sufficiently separate from the allowable range boundaries: the maxima lie either outside these boundaries or, in the case of the *L*_2w_ norm, very close to the zero concentration axes, making the solutions experimentally unfeasible.

**Figure 6:**
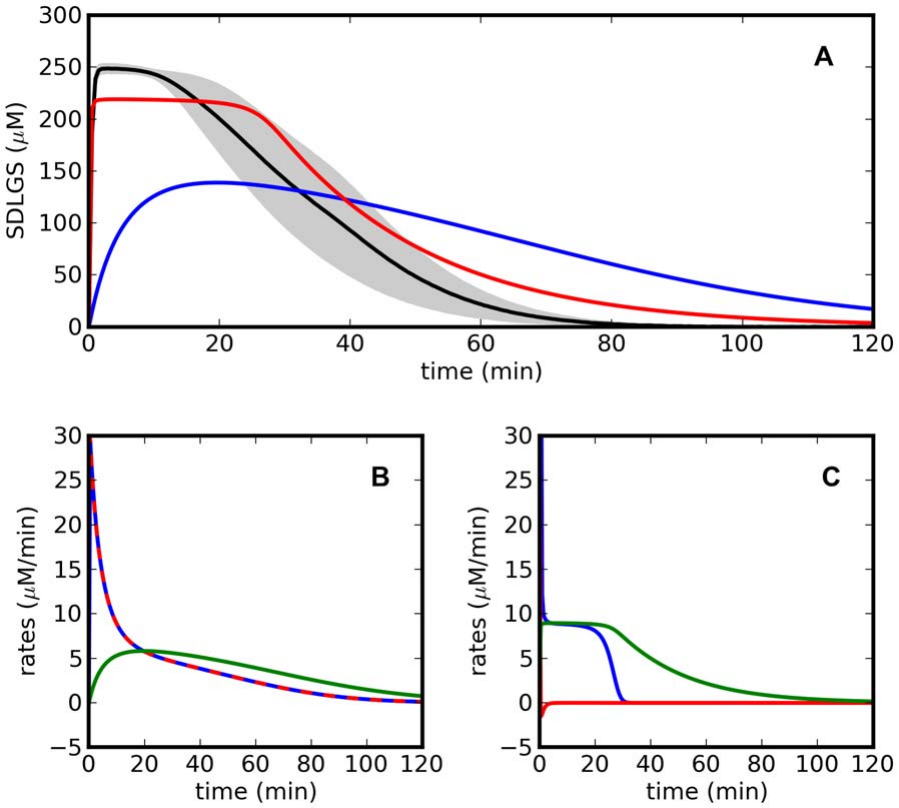
Discriminatory experiment for the kinetics of yeast glyoxalase I. A - Time courses of SDLGS concentration in the discriminatory setup experiment. Black: experimental result, average of 4 replicates (the grey shaded area is within one standard error of the mean). Blue: prediction by model 1. Red: prediction by model 2. Initial concentrations are 0.221 mM for glutathione, 2.0×10^−3^ mM for glyoxalase I, 0.441 mM for methylglyoxal and 4.0×10^−3^ mM for glyoxalase II. The initial concentrations correspond to the solution chosen from of the Pareto front highlighted in figure 4A. B and C - rates predicted by model 1 (B) and model 2 (C). Red: net rate of hemithioacetal formation, blue: rate of glyoxalase I reaction. green: rate of glyoxalase II reaction.

The time courses predicted by one of the solutions of figure 4 are shown in figure 6A (the time courses for the other solutions are very similar). For the first 120 min of reaction, approximately 4.5 fold less than the time necessary for parameter estimation (figure 3), the time courses of SDLGS predicted by each model are clearly divergent, both in concentration and rate of change.

It is interesting to clarify why in this example the optimal design concentrations are able to provide such a divergence between the time courses. In figure 6, panels B and C, the net rates of the different reactions are plotted against time and the explanation for the divergence becomes apparent: in model 1, the rates of the enzymatic reactions are limited by the non-enzymatic formation of the hemithioacetal and the net rate of this step is indistinguishable from the rate of glyoxalase I whereas, in model 2, the enzyme-catalyzed reactions are limited only by the regeneration of the cofactor glutathione and the hemithioacetal formation reaction is at *quasi* equilibrium throughout the time course.

The choice of the best model to describe the kinetics of the glyoxalase system was provided by actual laboratory experiments where the concentration of SDLGS was followed for 120 min, starting at the concentrations prescribed by the experimental design optimization (Figure 6 A). It is clear that only model 2 is able to predict the rapid initial increase of SDLGS concentration, followed by a short period of quasi steady state before decreasing to zero. The variation of the concentration of SDLGS predicted by model 1 is smoother and the decay to zero lasts longer. However, even model 2 does not describe completely well the early amplitude of SDLGS concentration, a fact that can be attributed to the discrepancies between the computed design and its experimental implementation associated with the experimental error in both enzyme and substrate concentrations. Nevertheless, it is clear that model 2 describes the experimental observations better than model 1.

It should be noted that the presence of glyoxalase II in the design is essential for the regeneration of this cofactor. Although the main goal of the discrimination concerns the kinetics of glyoxalase I, the occurrence of the non-enzymatic step, which is unavoidable and is not under the control of the experimenter, and the presence of glyoxalase II, which was deliberately added to the reaction network, provide the necessary degrees of freedom in the candidate models to support the design of a sufficiently complex experiment even in case where a single output variable is measureable. Also, it simulates the conditions found in total protein extracts and *in vivo*, where both enzymes are present and act simultaneously [30]. This is in contrast with previous studies on the kinetic characterization of glyoxalase I mechanism where classical initial-rate analysis was used and glyoxalase II was not present [31], [34]. Using only the initial-rate approach, the rate equation proposed for the porcine erythrocyte enzyme [31], for example, derives from a random mechanism and comprises six kinetic parameters. This equation might be over parameterized. Our findings suggest a simpler equation for the kinetics of glyoxalase I, a result that was achieved by working with full time courses and including another enzyme that provided a response from the system with discriminatory power. This approach is in line with the modern systems biology concepts of kinetically studying whole pathways and proposing models based on data that result from system perturbations that affect cellular networks as a whole [38].

In conclusion, the results of this work show that the combination of a multi-objective optimization algorithm with the extended Kullback-Leibler distance as objective function successfully provide experimental designs, within a reasonable computational time, to discriminate between two candidate models. This procedure may be useful for model construction in systems biology, where accurate models of biological processes are required. The difficult glyoxalase I discrimination problem, long addressed but not solved, was tackled with the proposed method and a model (model 2) was conclusively selected from a set of two candidates. The multi-objective approach presented in this paper has interesting potential to be explored in the future, due to the possibility of including additional objective functions in the optimization. Also, the approach is immediately usable for problems with more than two candidate models – for such cases, divergence between pair-wise combinations of models can be maximized simultaneously, as illustrated in figure 1B. Another interesting possibility is the addition of objective functions for experimental optimization for other purposes besides model discrimination. Experimental design for model discrimination and model parameter estimation are generally treated as distinct problems, and solutions for these two optimization problems tend to be different. The multi-objective approach may open a window to design experiments where a good compromise between optimization for model discrimination and parameter estimation is achieved.

## Materials and Methods

### Model details

The glyoxalase system, responsible for the elimination of methylglyoxal, a toxic and mutagenic byproduct of glycolysis [33], was chosen to validate the proposed method of experimental design. In the two models compared in this work (figure 2), glutathione and methylglyoxal undergo a non-enzymatic reversible condensation that results in a hemithioacetal. Mass-action kinetics was considered for this step, using previously published rate constants as in [29]. In model 1, the kinetics of glyoxalase I was described by the irreversible Michaelis-Menten equation with one substrate. In model 2, a sequential mechanism for two substrates was considered, using a simplified version of the irreversible two-substrate Michaelis-Menten equation [29]. In this simplification, the rate law is identical to the steady-state rate equation derived for this kind of mechanism except for the constant term in the denominator: this term is the product of the Michaelis constant of the second substrate with the inhibition constant of the first substrate as a product inhibitor of the reverse reaction [39]. Here assume that this constant term is the product of the Michaelis constants of the two substrates. This simplification eases the identification of the parameters of model 2 without loss of relevant information about the mechanism and the kinetic properties of the enzyme. The kinetics of glyoxalase II was described by the irreversible Michaelis-Menten equation with one substrate. The models were mathematical expressed by systems of ordinary differential equations. Model 1 is described by equations 7 to 10:
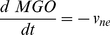
(7)

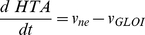
(8)

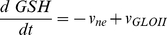
(9)

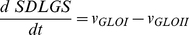
(10)


Model 2 is described by equations 11 to 14:
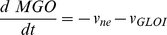
(11)

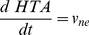
(12)

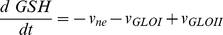
(13)

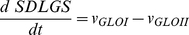
(14)


The rate laws assumed in the models are expressed in equations 15 to 18:
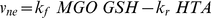
(15)

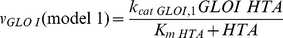
(16)

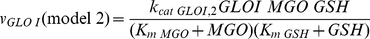
(17)

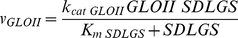
(18)


Apart from the non-enzymatic step, all parameters concerning the reactions catalyzed by enzymes were estimated as detailed below.

### Parameter estimation

Parameters of models 1 and 2 were estimated by collective fit to time-course data generated as follows: the reaction of glyoxalase I from *Saccharomyces cerevisiae* (Sigma) was monitored at 30°C in 70 mM potassium phosphate buffer pH 6.5 and the time course of SDLGS concentration was followed at 240 nm. Four time courses were generated by combining different concentrations of enzyme and the substrates glutathione (Roche) and methylglyoxal (figure 3 and dataset S1). Methylglyoxal was prepared fresh by heat-acid hydrolysis of methylglyoxal-1,1-dimethylacetal (Sigma) [40]. Reactions started with the addition of methylglyoxal to mixtures containing glyoxalase I and glutathione. The reaction of glyoxalase II from bovine liver (Sigma) was followed at the same temperature and pH. Four combinations of glyoxalase II and SDLGS (Sigma) concentrations were used to generate four different time courses of SDLGS hydrolysis (dataset S1). Absorbance was measured in an Agilent 8453 diode-array spectrophotometer with magnetic stirring and temperature control in the optical cells.

Since time-course parameter estimation poses the problem of fitting data to a set of non-linear ODEs, the use of stochastic optimization algorithms instead of deterministic algorithms is advised for their ability in finding global optima in multimodal functions [41], [42], [43], [44], [45].

Parameters were fitted using a (non-weighted) least-squares criterion, where the following objective, taken as a function of the vector of parameters **P**, was minimized:
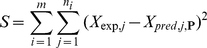
(19)
*m* is the number of time courses used in the estimation, *n_i_* is the number of experimental time points in time course *i*. *X_exp,j_* is the value of experimental SDLGS concentration at time point *j* and *X_pred,j_*_, **P**_ is the value predicted by either model at time point *j*, given the vector of parameters **P**.

This criterion was combined with the genetic algorithm Differential Evolution (DE) [46] coupled to the Downhill-Simplex algorithm. The initial population was generated by sampling a multivariate uniform distribution within a domain defined by constraints. These constraints are summarized in table S2.

Several recombination schemes have been proposed for use in DE, and the scheme called DE/rand/1/bin [41] was used with probability of replacement and the weighting factor for the combination of random vectors set to 0.7 and 0.5, respectively. This recombination scheme is the simplest proposed for use with differential evolution and has the advantage of keeping the population of candidates well distributed in the search space while converging to the optimal solution. The optimal solutions found by DE are further refined by the deterministic downhill-simplex algorithm [47] to improve the accuracy of the estimates [42].

The inverse of the Fisher information matrix was used as the parameter variance-covariance matrix, taking the square root of its diagonal as lower-bounds to parameter standard deviations [2]. The Fisher information matrix was computed as outlined in [2]:
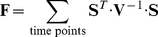
(20)
**V** is the measurement error variance-covariance matrix and **S** is the dynamic sensitivity matrix. The entries in this matrix can be computed by extending the model system of ODEs with the following differential equations [48]:
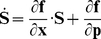
(21)
**x** is the vector of variables, **f** is the vector of the right end side of the model system of ODE and **p** is the vector of parameters.

### Experimental design optimization

Evolutionary algorithms are naturally suited for multi-objective numerical optimizations since the generation of sets of possible solutions allows finding several optimal solutions in a single run [17]. Also, they are generally less susceptible than other stochastic algorithms to be trapped at suboptimal approximations of the Pareto front [49]. In an evolutionary algorithm the successive selection of new or old candidate solutions to form the next generation may also be directly used as a way to approach the Pareto front through the dominance criterion [19].

Generalized Differential Evolution, presently in its third generation (GDE3) [19], was chosen as the multi-objective optimization algorithm. This is an extension of the single-objective optimization algorithm Differential Evolution. GDE3 performed well in a variety of problems both regarding computation time and distribution of the final solution set near the Pareto front [19], [50].

Initialization of the population, mutation and crossover in the GDE3 algorithm are identical to single-objective differential evolution. In the selection step, GDE3 is based on Pareto dominance and solution crowding: if a new solution vector dominates the target vector, the latter is replaced by the former in the new population. When the two solutions are non-dominated both are saved. As a consequence, after the evaluation of a set of new solutions the dimension of the population usually increases. To maintain population size during the progression of the algorithm, solutions were sorted according to Pareto dominance; then, surplus solutions were discarded according to proximity to other solutions (crowding) – one at a time, the solution closest to 3 other solutions was removed [19]. Solution vectors outside the user-defined boundaries were also discarded and generation of new vectors was repeated.

The most time-expensive step of GDE3 is the sorting of the non-dominated solutions, possibly surpassed by the evaluation of the objective functions only [50], [51]. To perform the non-dominated sorting, a *divide-and-conquer* mechanism based on a *dominance tree* data structure was employed.

In a dominance tree, nodes correspond to solutions and are interconnected through dominance or non-dominance relationships. The divide-and-conquer method builds the tree by consecutively *dividing* the entire population in halves, so that each node contains a single solution. The individual nodes are then successively compared pair-wise and merged (conquering) according to their dominance relationship until the dominance tree is complete. The algorithm is recursive in both the dividing and conquering steps since the result of dividing or merging nodes is subsequently used for other dividing or merging rounds. After sorting, the solutions are organized in sets named non-dominated fronts; these sets are ranked such that the solutions of any front are non-dominated by other solutions of the same front and there is at least one solution in front *i* +1 dominated by at least one solution in front *i*.

After sorting, the most crowded solutions of the last non-dominated front were iteratively removed from the population to restore its original size. In the present implementation of GDE3, the *k*-nearest neighbor method [19], [52] was used to identify the most crowded solutions in the last non-dominated front.

The termination criterion for the optimization was defined as non-improvement in more than 5% of the possible solutions for 20 generations of GDE3. In addition, a maximal number of generations was set to 200.

### Computational implementation

The computational algorithms were implemented in a software package (S-timator) written in Python (www.python.org) and using the modules *numpy* (numpy.scipy.org), and *scipy* (www.scipy.org) for numerical efficiency. The module *sympy* (http://code.google.com/p/sympy) was used for symbolic derivation of dynamic sensitivities and the module *matplotlib* (http://matplotlib.sourceforge.net/) for plotting. The *odeint* function from the *scipy.integrate* module, which implements the LSODA routine [53], [54] was used for ODE numerical integration. All the source code used for the computations performed in this work is available from http://enzymology.fc.ul.pt/software.

## Supporting Information

Dataset S1 **Time courses used in parameter estimation.** The dataset includes four time courses used in the estimation of glyoxalase I parameters and four time courses used in the estimation of glyoxalase II parameters.
(TXT)

Table S1 **Optimization boundaries used in experimental design.**
(DOC)

Table S2 **Optimization boundaries used in parameter estimation.**
(DOC)
