# Supplementary material for: Optimization of Time-Course Experiments for Kinetic Model Discrimination
Source: PLoS One. 2012 Mar 5;7(3):e32749. doi: 10.1371/journal.pone.0032749 (PMC3293846; doi:10.1371/journal.pone.0032749)
Supplement: Table S1 — Optimization boundaries used in experimental design. (DOC) [file pone.0032749.s002.doc]

**Table S2.** **Optimization boundaries used in experimental design.**

| **Variable** | **Lower** | **Upper** |
| --- | --- | --- |
| Glyoxalase I concentration | 0 | 2.0×10^-3^ mM |
| Glyoxalase II concentration | 0 | 4.0×10^-4^ mM |
| Initial GSH concentration | 0 | 1 mM |
| Initial MGO concentration | 0 | 1 mM |
