# Supplementary material for: Optimization of Time-Course Experiments for Kinetic Model Discrimination
Source: PLoS One. 2012 Mar 5;7(3):e32749. doi: 10.1371/journal.pone.0032749 (PMC3293846; doi:10.1371/journal.pone.0032749)
Supplement: Table S2 — Optimization boundaries used in parameter estimation. (DOC) [file pone.0032749.s003.doc]

**Table S3. Optimization boundaries used in parameter estimation.**

| **Enzyme** | **Parameter** | **Lower** | **Upper** |
| --- | --- | --- | --- |
| **Glyoxalase I, model 1** | *k*_cat GLOI,1_ | 10^3^ min^-1^ | 10^5^ min^-1^ |
|  | *K*_m,HTA_ | 0.05 mM | 1 mM |
| **Glyoxalase I, model 2** | *k*_cat GLOI,2_ | 1 ×10^5^ min^-1^ | 2 ×10^5^ min^-1^ |
|  | *K*_m,GSH_ | 0.05 mM | 1 mM |
|  | *K*_m,MGO_ | 0.1 mM | 5 mM |
| **Glyoxalase II** | *k*_cat GLOII_ | 10^2^ min^-1^ | 10^3^ min^-1^ |
|  | *K*_m SDLGS_ | 0.05 mM | 10 mM |
